# A clinician’s guide to understanding aortic 4D flow MRI

**DOI:** 10.1186/s13244-023-01458-x

**Published:** 2023-07-03

**Authors:** Mitch J. F. G. Ramaekers, Jos J. M. Westenberg, Bouke P. Adriaans, Estelle C. Nijssen, Joachim E. Wildberger, Hildo J. Lamb, Simon Schalla

**Affiliations:** 1 Department of Cardiology and Radiology and Nuclear Medicine, Maastricht University Medical Center +, P. Debyelaan 25, 6229 HX Maastricht, The Netherlands; 2 Cardiovascular Research Institute Maastricht (CARIM), Maastricht, The Netherlands; 3 Department of Radiology, Leiden University Medical Center, Albinusdreef 2, 2333 ZA Leiden, The Netherlands; 4 Department of Radiology and Nuclear Medicine, Maastricht University Medical Center +, P. Debyelaan 25, 6229 HX Maastricht, The Netherlands; 5 Department of Cardiology, Maastricht University Medical Center +, P. Debyelaan 25, 6229 HX Maastricht, The Netherlands

**Keywords:** 4D flow MRI, MRI, Aorta, Blood flow, Vessels

## Abstract

Four-dimensional flow magnetic resonance imaging is an emerging technique which may play a role in diagnosis and risk-stratification of aortic disease. Some knowledge of flow dynamics and related parameters is necessary to understand and apply this technique in clinical workflows. The purpose of the current review is to provide a guide for clinicians to the basics of flow imaging, frequently used flow-related parameters, and their relevance in the context of aortic disease.

**Clinical relevance statement** Understanding normal and abnormal aortic flow could improve clinical care in patients with aortic disease.

**Graphical abstract:**

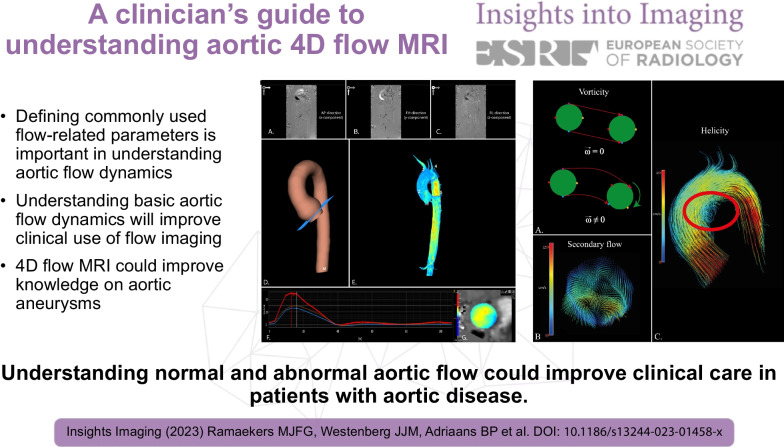

## Key Points

- Defining commonly used flow-related parameters is important in understanding aortic flow dynamics.
- Understanding basic aortic flow dynamics will improve clinical use of 4D flow imaging.
- 4D flow MRI could improve knowledge on aortic aneurysms.

## Introduction

Conventional two-dimensional (2D) phase contrast MR imaging has long been known and used in clinical practice, to quantify intra-cardiac shunting or aortic valve regurgitation for example [1, 2]. 2D phase contrast imaging generally is two dimensional in a tomographic plane (x and y axis) and includes time-resolved through plane velocity data (one-directional). Three-dimensional (3D) phase contrast imaging uses a third dimension, or z-axis, on top of the two dimensions, or x- and y-axes, already present in 2D phase contrast imaging, enabling measurement of three-directional flow. Four-dimensional (4D) flow imaging incorporates time as fourth dimension and is based on time-resolved 3D and three-directional phase contrast imaging. Thus, 4D flow magnetic resonance imaging (MRI) is an imaging technique from which both qualitative and quantitative data on hemodynamic properties can be obtained.

Even though 4D flow MRI is increasingly used in the clinical setting to assess pathophysiology of congenital heart disease, it is primarily known from areas of research [3, 4]. The technique has been of key interest for investigating flow profiles in the heart, aorta, and pulmonary artery. Mounting evidence suggests a link between aberrant flow and aortic pathology, such as a relationship between disturbed flow and progression of aortic aneurysms [5, 6].

The blood flow velocity vectors, comprised in 4D flow MRI data (i.e., direction and magnitude over time), can be used to generate reconstructed and time-resolved images, showing colorful representations of flow-patterns. The raw data enable calculation of an abundance of flow-related parameters, which allow more accurate descriptions of hemodynamics. Wall shear stress (WSS), rotational parameters such as vorticity and helicity, and distribution or loss of kinetic energy are examples of variables critical to the assessment of blood flow. However, results of in-depth analyses of flow images are not easily understood.

This narrative review aims to help clinicians understand detailed analyses of aortic flow imaging. To this end, the first section gives an overview of image acquisition, including an explanation of general flow-dynamics and 4D flow MRI examples. Subsequent sections detail the various flow-related parameters and their relevance in the context of aortic disease.

## Acquisition of flow data

Besides using 4D flow MRI, images depicting in vivo blood flow can be acquired using ultrasonography (US), and flow can be modeled using computational fluid dynamics (CFD). Doppler US is the most basic technique enabling measurement of flow velocities and directions, but it is limited to retrieving 3D flow patterns and flow-related parameters. Furthermore, measurement of aortic peak velocities using Doppler US could result in lower values compared to 4D flow MRI in patients with aortic valve stenosis[2]. US stereoscopic particle image velocimetry has shown potential as a tool for obtaining 4D velocity data in the aorta and left ventricle [7, 8]. Computed tomography (CT), often applied in serial imaging studies of patients with aortic aneurysms, does not currently allow direct imaging of flow. However, CT can be helpful in calculating flow-related parameters by combining anatomical information with CFD [9].

CFD is a modeling method that enables calculation of all flow-related parameters based on 3D/4D geometry and inlet and outlet flow [10]. Inlet and outlet flow can be acquired from 2D flow MRI, echo Doppler, or simply by applying certain assumptions. Flow within the provided geometry can then be simulated by incorporating these boundary conditions into the Navier–Stokes equations, the governing equations of mass and energy conservation for an incompressible fluid flow in a closed system. Even though the solution of the Navier–Stokes equations obtained using CFD is always an approximation based on modeling assumptions, the spatial and temporal resolution that can be achieved, especially when using fine meshes, is far superior to that achieved using imaging techniques such as MRI or echo Doppler. However, a major disadvantage of CFD is the long and complex computational processing, requiring step-by-step manual input including 3D segmentation and model optimization for mathematical analysis [11].

### MRI acquisition and MR systems

The acquisition of 4D flow MRI is based on phase contrast imaging (PC MRI). In PC MRI, two bipolar gradients are used to modulate the phase signal of moving protons. Therefore, PC MRI can evaluate protons moving through a single plane (2D), or through 3D space resulting in velocity data. Traditionally, PC MRI has been used on various vessels, for instance the aorta, to obtain unidirectional 2D flow maps from cross-sectional slices [12], but acquiring 3D and three-directional flow maps, and thus 4D flow MRI, is also possible [13]. According to the prevailing consensus statement, the recommended spatial resolution for aortic 4D flow MRI is 2.5 × 2.5 × 2.5 mm or smaller, and the aim for temporal resolution is 30 ms or shorter [14]. Ideally, a higher temporal and spatial resolution (if isotropic) is achieved, especially in congenital 4D flow MRI. The acquisition time is between 5 and 25 min. 4D flow MRI can be acquired without contrast agents, but use of contrast agents improves the velocity-to-noise ratio (VNR) and distinction from surrounding tissues. It must be noted, however, that contrast agents may wash out during 4D flow acquisition, potentially influencing blood T1 times, i.e., the time it takes blood to return to its equilibrium state after excitation [14]. The flip angle is recommended to equal the Ernst angle, but in practice a slightly higher angle is often chosen because of in-flow effects. Electrocardiogram and respiratory gating are recommended to reduce motion artifacts. Velocity encoding (VENC) needs to be equal to or greater than the expected peak velocity, but as close to the peak velocity as possible, to achieve maximum VNR: VENC set too low leads to areas with aliasing, which must be corrected (phase-unwrapping) before being used in quantification during post-processing; VENC set too high leads to lack of VNR and contrast and thus lack of quality and accuracy. The multi-VENC approach is a promising new technique which leads to better representation of both regions with high and low velocity (i.e., optimal dynamic range), but it has a trade-off in terms of longer scan times and a lower temporal resolution [15, 16].

4D flow MRI can be obtained on all systems, including 1.5, 3 and 7 Tesla (T) [17–19]. Between these systems, an increase in signal to noise ratio has been observed, a factor 1.7 increase from 1.5 to 3 T and a factor 2.2 increase from 3 to 7 T, resulting in improved image quality with increasing field strength [18].

### Limitations of 4D flow MRI in aortic disease

4D flow MRI has some limitations, such as longer acquisition time and lower spatial- and temporal resolution. It is expected that scan duration of 4D flow MRI will be shortened in future due to faster scanning techniques (e.g., using compressed sensing) [20]. 4D flow MRI voxel size is relatively coarse, and temporal resolution is less than that achieved using 2D flow. 

In large aneurysms, variations in velocities may occur due to flow displacement (explained in more detail later in the text), but this may also be a problem with 2D flow imaging. On the other hand, 4D flow enables a multi-VENC approach as described above.

Post-processing of 4D flow MRI is more complex than 2D flow imaging, although post-processing time as well as user-dependency can be reduced by automating segmentation and quantification processes and deep-learning [21, 22]. Quantitative 4D flow analysis is further complicated by the fact that data on normal or reference values are currently limited, and values may vary according to vendor systems and centers [23, 24]. Finally, 2D and 4D flow MRI are both hampered by metal artifacts such as prosthetic valves. More detailed overviews of disadvantages/advantages of 4D over 2D flow MRI can be found in literature [4, 25].

### MRI data analysis

**Fig. 1:**
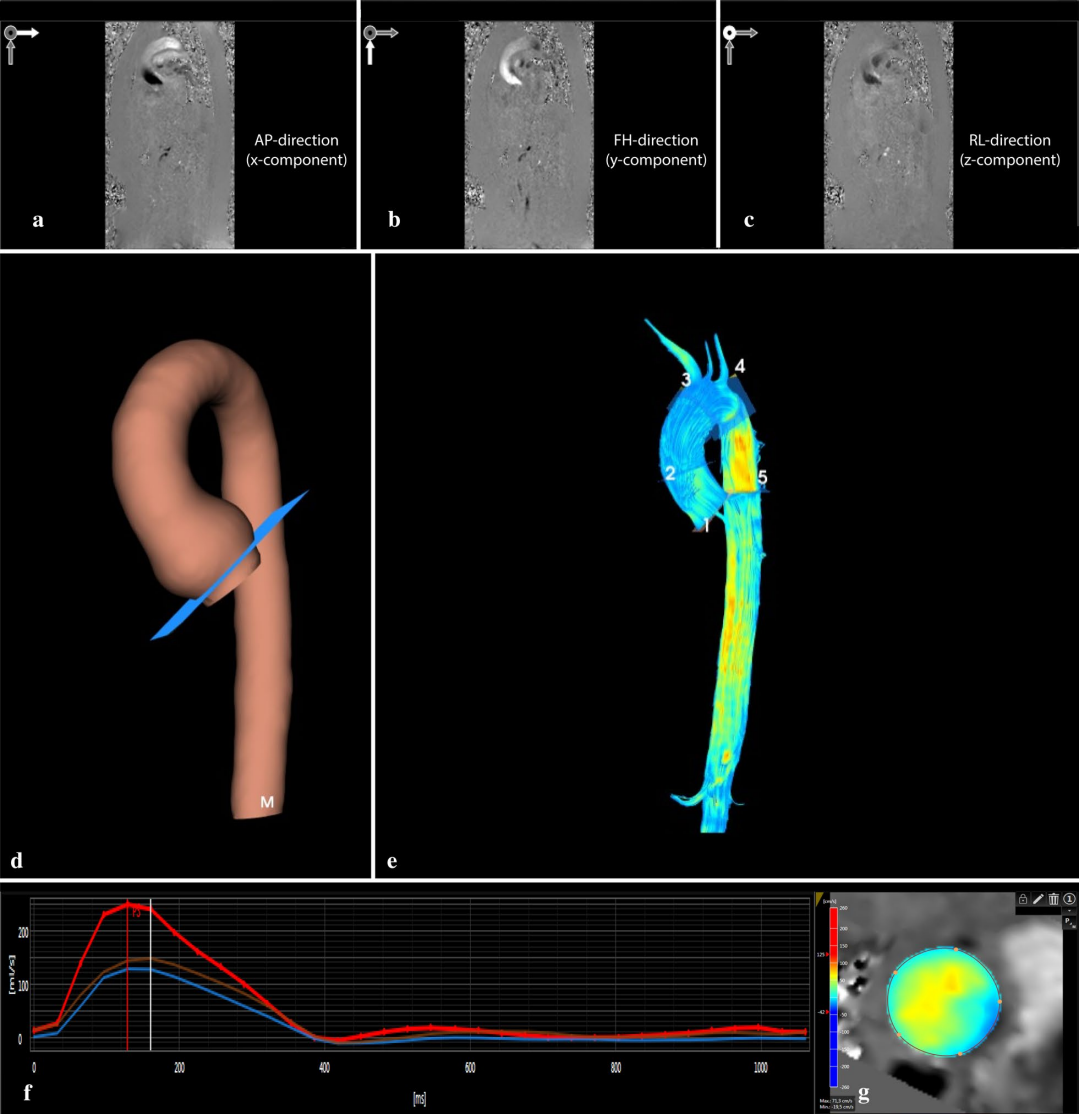
General workflow of analyzing aortic 4D flow MRI data. **a**–**c** depict the phase images of the phase contrast data with velocity encoding in three directions (i.e. anterior–posterior (AP; X-component), feet-head (FH; Y-component), and left–right (LR, Z-component). After the pre-processing steps, including phase offset and anti-aliasing correction, luminal segmentation is performed on the combined phase contrast images. This results in a 3D model as seen in image **d**. Image **e** represents the streamlines of blood flow within the aortic volume. Manual placement of 2D planes along the centerline results in a quantified flow graph (**f**). Each line represents a 2D plane and a cross-sectional image of through plane velocity profile (**g**). Red depicts high velocity; blue depicts low and retrograde velocity

**Fig. 2:**
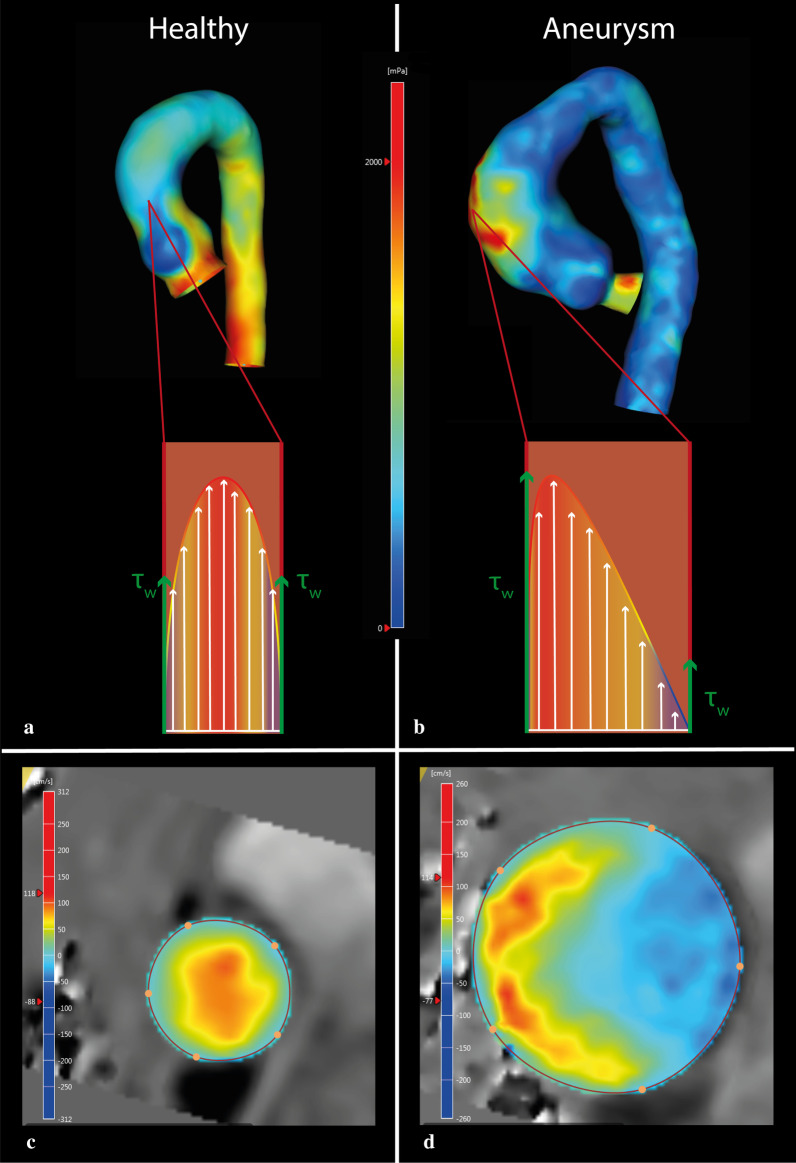
Distribution of wall shear stress in the thoracic aorta of a healthy control (**a** and **c**) and a patient with an aneurysm of the ascending aorta (**b** and **d**). The schematic image illustrates a laminar-like flow pattern in **a**, with high velocities in the center of the vessel and low velocities along the vessel wall. In **b**, the schematic image shows a skewed flow profile with highest velocities away from the center of the vessel. In the healthy volunteer (**a**), an even distribution of WSS (*τ*_w_) is seen, whereas the patient with the aneurysm (**b**), shows higher WSS at the anterior side of the ascending aorta and lower WSS on the opposite side. **c**, **d** depict corresponding cross-sectional flow profiles. **c** (healthy control) depicts a flow pattern with almost no flow displacement, whereas **d** (degenerative ascending aortic aneurysm) depicts a flow pattern with large flow displacement. **a** and **b** Red depicts high WSS; blue depicts low WSS. **c** and **d** Red depicts high velocity; blue depicts low and retrograde velocity

Flow rates and velocity profiles form the basis of all 4D flow related calculations. The processing steps are illustrated in Fig. 1 and depend on the type of analyzing software used. First, data must be corrected for several known errors: phase-offset errors associated with eddy currents and gradient terms require phase-offset correction [26], and any areas with aliasing need to be corrected by using phase unwrapping, also known as anti-aliasing [27]. Data analysis starts with phase images generated from the phase contrast source data, with velocity encoding along the three axes (x, y, z), also referred to as the feet-head (FH), left–right (LR), and anterior–posterior (AP) directions (Fig. 1A–C). These images are then combined to create a 3D image; this can be used in luminal segmentation of the aortic volume for each acquired cardiac phase to generate a 3D model for anatomical reference. Within this 3D aortic model, qualitative assessment is possible by visualizing flow streamlines (Fig. 1D, E) and mapping of wall shear stress (WSS) (Fig. 2A, B). Within this 3D volume quantitative assessment can be performed by creating a plane perpendicular to the centerline of the vessel, from which through-plane flow rate, velocity, and WSS data can be extracted. These and other advanced parameters are detailed below and summarized in Table 1.

**Table 1:** Flow-related parameters

Flow parameters	Short definition	Unit
Flow rate	The amount of blood passing through a plane per time unit (also known as through-plane volume per second)	mL/s
Flow volume	Volume of blood passing through a plane within a certain amount of time. Derived from the area under the curve of the flow rate graph (flow [ml/s] versus time [s])	mL
Flow velocity vector	Magnitude and direction of blood flow through a plane (also known as through-plane propagation speed of blood)	m/s or cm/s
Flow jet angle	The angle between the direction of blood flow and the centerline of the vessel	° (degrees)
Flow displacement	The degree of eccentric flow, defined as the distance between the center of the vessel and the center of velocity (often normalized to lumen diameter)	mm (or relative to lumen diameter)
Vorticity	Local in-plane rotation of a fluid particle around a common axis (comparable to the angular velocity of solid objects)	1/s
Helicity	Alignment of vorticity with the main velocity vector	m/s^2^
Wall shear stress	The tangential force caused by friction of blood as it flows along the vessel wall	Pascal or dyne/cm^2^
Kinetic energy	The energy that is stored in movement of blood	Joules
Viscous energy loss	The energy that is lost due to frictional forces	Joules
Turbulent kinetic energy	The energy that is stored in turbulent flow of blood	Joules
Pulse wave velocity	The propagation speed of a pulse wave created by the systolic contraction of the left ventricle (a measure of arterial stiffness)	m/s
Pressure fields	Differences in distribution of local pressure within a vessel (gradients between local pressure fields are used to assess hemodynamic significance of obstructions)	mmHg or Pascal

## Flow rate, flow volume, flow velocity, flow jet angle and flow displacement

These parameters can be quantified directly from the phase-contrast images at any location within the volume of interest. Flow rate is defined as the amount of blood passing through a plane per time unit. Flow rate is mostly presented in milliliters per second and peaks during systole for the aorta and pulmonary artery, and during diastole for the mitral- and tricuspid valve.

The area under the curve of the flow rate graph (flow [ml/s] versus time [s]), represents the flow volume in milliliters. Net flow volume is generally positive, defined as the sum of both antegrade (positive) and retrograde (negative) flow volumes at different points in time. At the level of the aortic valve, antegrade flow volume during one cardiac cycle is equal to left ventricular stroke volume (in absence of valvular regurgitation or intracardiac shunting). This variable is known from cardiac physiology: the product of stroke volume and heart rate determines cardiac output. Measurement of retrograde flow and calculation of the regurgitation fraction can be used to quantify aortic valve regurgitation [28].

Flow velocity is related to flow rate, but flow velocity is a vector with both magnitude and direction. The velocity magnitude describes the speed of the blood in (centi)meters per second. Its value depends, among other factors, on blood pressure, stroke volume, aortic valve area, and vascular resistance. A typical example of abnormal high flow velocities is in severe aortic valve stenosis which is linked to aortic dilatation (post-stenotic dilatation) [2].

Besides flow velocity magnitude, direction may provide important information. Aortic flow moves from proximal to distal, starting at the aortic valve. In efficient flow, the center of velocity is typically aligned with the center of the vessel lumen, with flow velocity decreasing closer to the vessel wall (Fig. 2A, C). This means that the profile of flow velocity vectors is parabolic, also called a laminar pattern or Womersley profile. The Reynolds number is used to express the ratio between the product of flow velocity, blood density and diameter on one hand, and blood viscosity on the other. When this number increases, the flow velocity profile can become turbulent, consisting of chaotic flow instabilities. This means that a fluctuating component in random direction is introduced, which affects the direction of each respective velocity vector. In other words, flow is laminar when the Reynolds number is low, and becomes turbulent when the Reynolds number is high [29]. If the Reynolds number remains below 2300, flow remains laminar even if the geometry changes from a straight tube to a U-bend, such as the aortic arch.

**Fig. 3:**
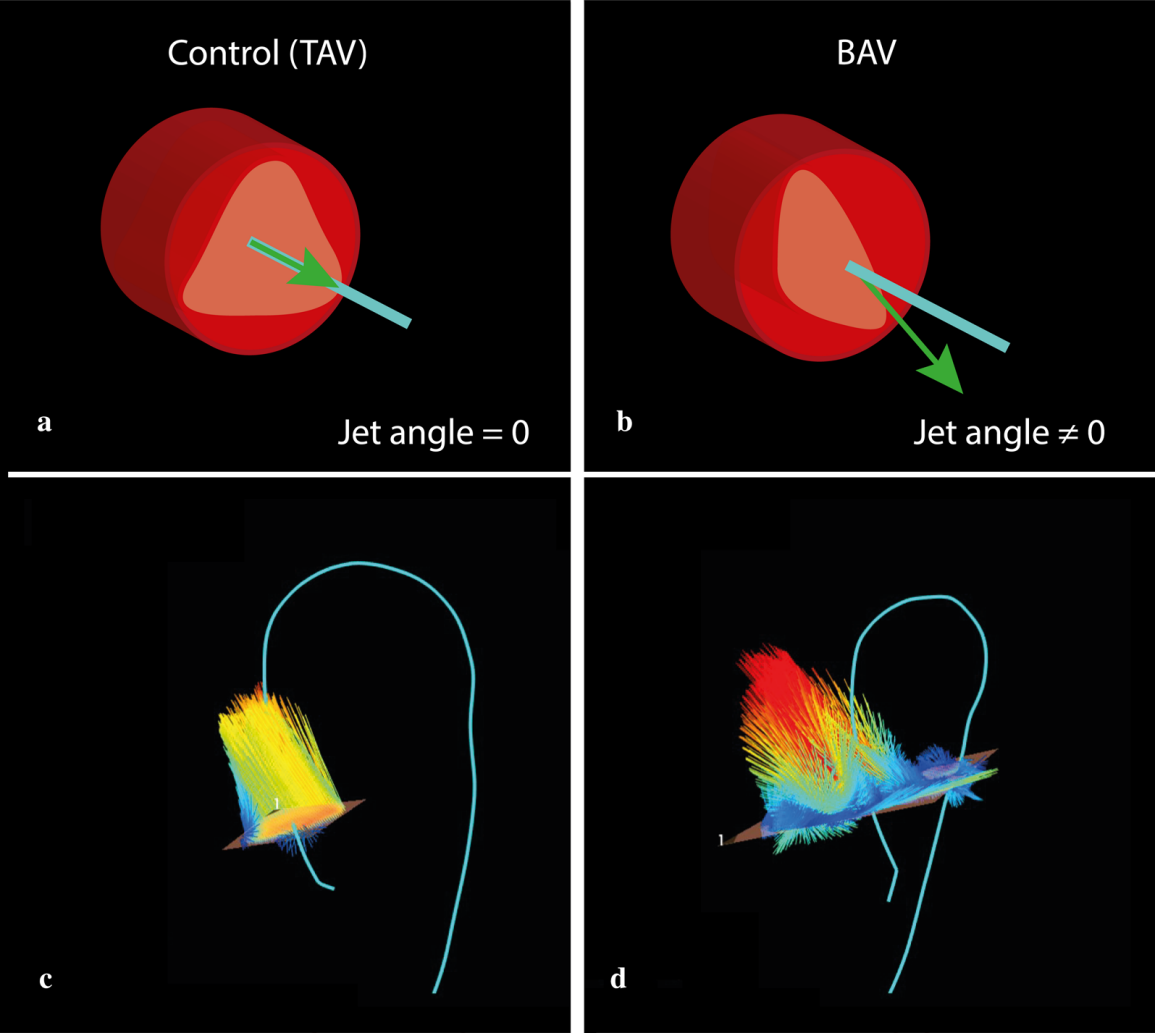
Schematic representations of normal and abnormal aortic jet angles (between the centerline and the direction of the velocity vector). **a** A normal jet angle (≈ 0°), with normally functioning tricuspid aortic valve: the green arrow, representing the velocity vector, is perfectly aligned with the light blue line, representing the geometric centerline of the aorta. **b** Abnormal jet angle (≠ 0˚), with bicuspid aortic valve (BAV): the velocity vector is misaligned with the aorta centerline. **c** Velocity vectors at the level of the aortic annulus with a normal flow jet angle: the vectors are generally aligned to the (light blue) centerline. **d** Velocity vectors at the level of the aortic annulus with abnormal flow jet angle: the vectors are generally pointed away from the centerline and thus are directed towards the aortic wall

During systole, the left ventricle will create enough positive pressure to open the aortic valve, which initiates aortic flow. This will start to develop into laminar flow, provided that the Reynolds number is low enough, and full development into laminar flow will follow provided there is sufficient inlet length. The distance between the aortic valve and aortic arch is insufficient to fully develop a laminar flow pattern in the ascending aorta, but flow in the latter can normally be considered near laminar. However, in some cases, flow in this trajectory can become eccentric, with the center of velocity shifting away from the center of the vessel lumen (Fig. 2B, D). The presence of an abnormal flow jet angle, defined as the angle between the centerline of the vessel and the velocity vector direction, can induce eccentric flow. Usually, this angle is close to zero (Fig. 2A, C). However, an increased flow jet angle is often caused by a non-symmetrical alteration of the aortic valve area, such as is the case in a bicuspid aortic valve (BAV), where the direction of the jet depends on the valve fusion phenotype (Fig. 3) [30].

The degree of eccentric flow can be quantified as flow displacement (FD), defined as the distance between the center of the vessel and the center of velocity (Fig. 3B, D). Increased FD has been linked to the development of aortic aneurysms and accelerated aneurysm growth rates in patients with BAV [31–33].

## Wall shear stress

Wall shear stress (WSS) is the tangential frictional force of viscous blood in the boundary layer, acting on the vessel wall in the direction of the blood flow. It is a vector with magnitude and direction, and can be separated into axial and circumferential vector components. 4D flow MRI can be used to map local WSS (Fig. 2A, B). However, true WSS is calculated in the sub-millimeter boundary layer near the vessel wall, therefore the limited spatial resolution of 4D flow MRI may underestimate WSS. The accuracy of WSS determined by 4D flow MRI is currently not well known [34–38]. In contrast, CFD can achieve WSS calculations at submillimeter resolution, but results are based on modeling assumptions. Ultimately, both 4D flow MRI and CFD can only provide estimates of local WSS rather than precise measurements [37]. However, several studies have shown that discriminating between areas of high and low WSS is sufficient to predict aortic remodeling, especially when similar scanning protocols are used [39–41] (Table 2).

**Table 2:** Flow deviations associated with different aortic diseases

Diseases	Associated flow parameters*
Acute aortic syndromes	↓ WSS (only in AAA)
Aortic aneurysm	↑ WSS, circumferential WSS
	↓ Vorticity and helicity
	↑ EL (in vitro studies)
Aortic valve regurgitation	↑ Regurgitation fraction
	↑ Retrograde flow
Aortic valve stenosis	↑ Flow velocity
	↑ KE, EL, and TKE
	↑ Pressure gradient
Atherosclerosis	↓ Vorticity and helicity
	↓ WSS
	↑ Pulse wave velocity
Bicuspid aortic valve	↑ Flow velocity (if accompanied by AoS)
	↑ Flow displacement and flow jet angle
	↑ WSS
	↑ Vorticity and helicity
	↑ EL
Coarctation	↑ Flow velocity
	↑ Pressure gradient
Vessel wall stiffening	↑ Pulse wave velocity

*WSS* wall shear stress, *AAA* abdominal aortic aneurysm, *EL* energy loss, *KE* kinetic energy, *TKE* turbulent kinetic energy, *AoS* aortic valve stenosis

*Based on the literature as cited in the respective sections

The endothelial cells aligning vessel walls are constantly subjected to WSS, and any deviation in WSS initiates a biochemical response. For example, increased WSS causing vascular remodeling may cause aortic dilatation [41], and low and oscillating WSS at bifurcations is known to play a role in atherosclerosis [42].

In patients with BAV, dependent on which fusion phenotype is present, areas of high WSS are common due to the abnormal jet angle present in these patients [43, 44]. These areas of high WSS lead to degradation of extracellular matrix, particularly a decrease in elastin [40, 41]. A recent longitudinal study in patients with BAV found increased aortic growth rates in areas with high total as well as high circumferential WSS, obtained from 4D flow MRI [45]. In contrast, increased WSS has not been directly associated with increased aortic growth rates in subjects with tricuspid aortic valves. Some studies suggest a link between increased WSS and ascending aortic aneurysms, and an especially high circumferential WSS may be an important predictor of aneurysm formation [33, 45]. In thoracic aortic aneurysm (TAA) patients, local WSS maps beyond peak systole show a significant increase in WSS in the outer curvature compared to controls (Fig. 2B) [46]. This increase in local WSS is related to vessel wall thinning, extracellular matrix degradation, and loss of vascular smooth muscle cells [39]. Another study describes an increase in WSS in the outer curvature of a smaller heart-aorta angle (the angle between left ventricular outflow tract and left ventricle), suggesting this angle may be a cause for aberrant WSS distribution [47].

Whereas high flow conditions in the ascending aorta prevent atherosclerosis, lower flow conditions and areas of recirculating flow in the aortic arch and the abdominal aorta make these areas more prone to develop atherosclerosis. Indeed, low WSS and oscillatory WSS (often analyzed together) are inevitably linked to atherosclerosis [48, 49]. Neither is directly linked to aortic dilatation [50], but low WSS and subsequent thrombus formation have been linked to abdominal aortic aneurysm rupture [51].

## Vorticity and helicity

**Fig. 4:**
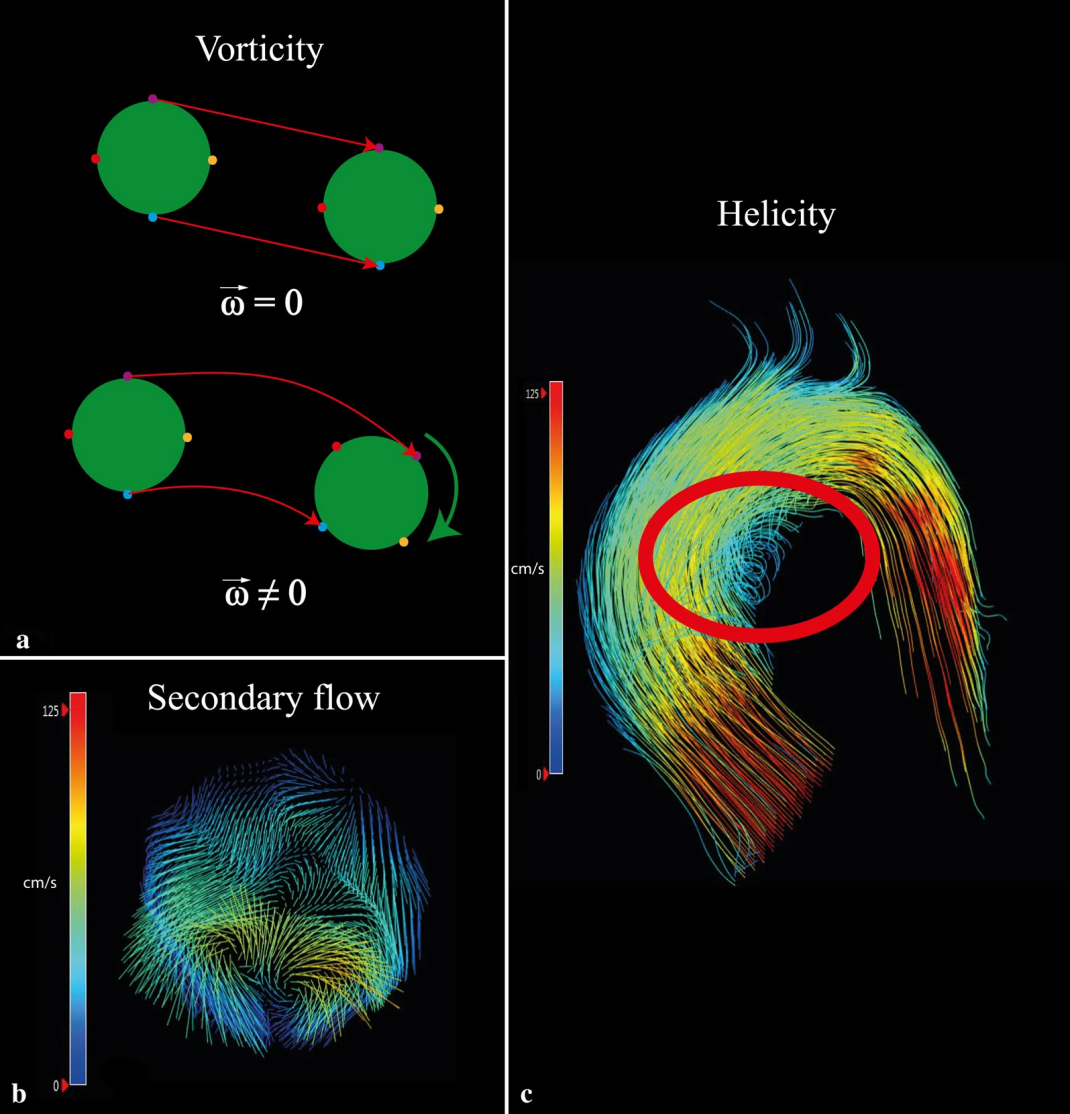
Schematic and in vivo visualization of rotational flow parameters (vorticity and helicity). **a** A schematic representation of vorticity ($\underset{\omega }{\to }$). The upper particle moves in space without rotating. The bottom particle moves in the same direction but also spins around its own axis, resulting in a curl. **b** Vorticity is clearly seen when looking at the in-plane vectors. This plane is located in the aortic arch where secondary flow patterns are physiological. In this case, two counter-rotating vortices are seen. **c** An example of helical flow (red circle)

Vorticity and helicity are two parameters describing rotational movement in flow patterns. Vorticity describes the local in-plane rotation of a fluid particle around a common axis and is comparable to the angular velocity of solid objects (Fig. 4A): particles move in space towards a certain direction, but also rotate around their own axis, resulting in a curl. Helicity is calculated from vorticity and the direction-determining main flow velocity component. It represents the alignment of the rotation to the main velocity component and can be right-handed (positive) or left-handed (negative) (Fig. 4C); both are general principles in flow dynamics.

Both physiological and pathological flow in the aorta are associated with rotational flow patterns. Leonardo Da Vinci already drew vortices in the aortic sinuses in the 1500 s; these were later confirmed using 4D flow MRI [52]. Rotational flow patterns can be visualized using pathline of 4D flow MRI, which show the virtual path of blood flow over time[53]. Normal rotational flow, as described by Kilner et al., shows a helical flow pattern towards end-systole, leading to preservation of laminar-like flow in the aortic arch [54]. Abnormal rotational flow patterns, as seen in some diseases such as BAV and degenerative aortic aneurysms [55–58], generally consist of areas where blood flow spins around an axis perpendicular to the blood flow (vortex), or around an axis parallel to the flow direction (helix). Visual grading studies found more pronounced presence of such vortices and helices in ascending aortic aneurysms [57, 59]. This leads to the expectation that vorticity and helicity will also be greater, as is the case in BAV [60], but quantified vorticity and helicity is actually less in patients with degenerative ascending aortic aneurysms compared to controls [46]. Indeed, Liu et al. describe the necessity of vorticity and helicity in stable blood flow and in the prevention of atherosclerosis [61, 62]: some vorticity and helicity levels are needed to maintain stable flow through aorta curves. Thus, both low and high levels of vorticity and helicity can be considered abnormal and may predispose to aortic disease.

## Kinetic energy, viscous energy loss and turbulent kinetic energy

**Fig. 5:**
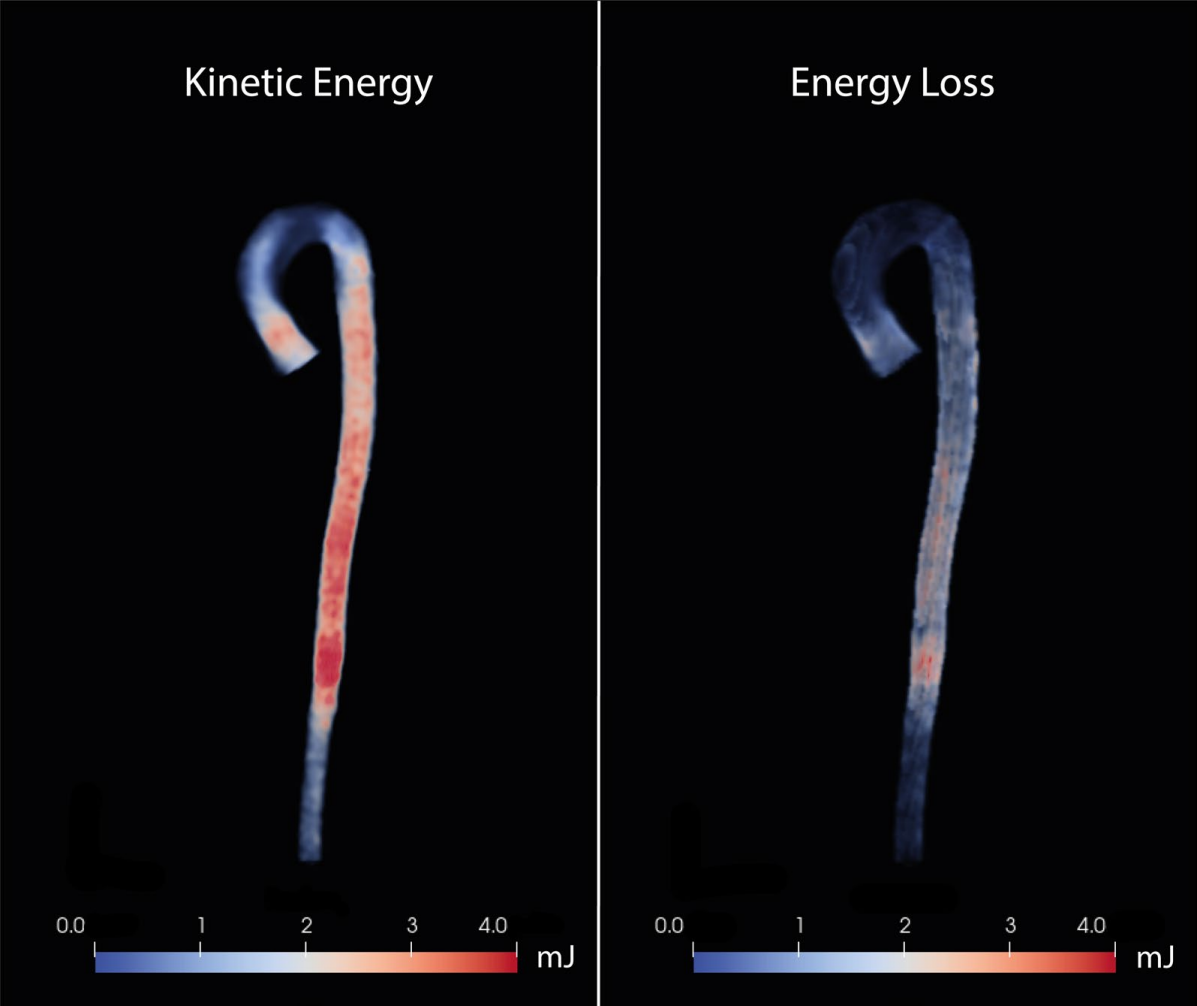
Color maps of kinetic energy (left) and viscous energy loss (right) in the total aorta in a healthy volunteer at peak systole (in millijoules [mJ])

Kinetic energy (KE), viscous energy loss (EL), and turbulent kinetic energy (TKE) are energy-related parameters. Kinetic energy is the amount of energy stored in the movement of mass. If movement is present, kinetic energy is present. In an ideal closed circuit, kinetic energy is fully preserved. However, viscous friction against vessel walls and between blood layers cause irreversible dissipation of kinetic energy, expressed as energy loss [63, 64]. Both kinetic energy and energy loss can be calculated from the velocity data acquired using 4D-flow MRI (Fig. 5).

Energy loss is a normal principle in physiologic blood flow. However, increased energy loss may have two nefarious consequences. First, the excess energy that is lost will be transferred to the vessel wall. Second, in response to the energy loss, afterload will be increased to maintain sufficient kinetic energy and pressure [65]. In vitro models found increased energy loss in a dilated geometry, and it is proposed as a possible indicator for aneurysm severity [66]. In-vivo studies show that energy loss is significantly increased in the ascending aorta in patients with TAA and aortic valve stenosis (with and without BAV) [63].

In certain situations, laminar flow can become turbulent. In turbulent flow, blood particles do not move coherently but are in chaotic motion instead, resulting in part of the kinetic energy of the system not contributing to efficient flow. The energy associated with the turbulent motion, TKE, can be quantified using 4D flow MRI [67, 68]. In simple terms, TKE is associated with the velocity range measured within a small volume and can be derived from the intra-voxel standard deviation of velocity vectors. This standard deviation of the velocity represents the fluctuation of velocity and is a commonly used measurement of turbulence [69]. The additional value of TKE in aneurysm assessment and prognosis is yet to be proven [66, 70].

In short, increased EL or TKE are pathological and do not contribute to efficient flow. Inefficient flow also affects left ventricle afterload. Thus, quantification of EL and TKE could provide valuable information on aortic valve stenosis and its influence on the left ventricle, especially in cases where echocardiography does not suffice.

## Pulse wave velocity

**Fig. 6:**
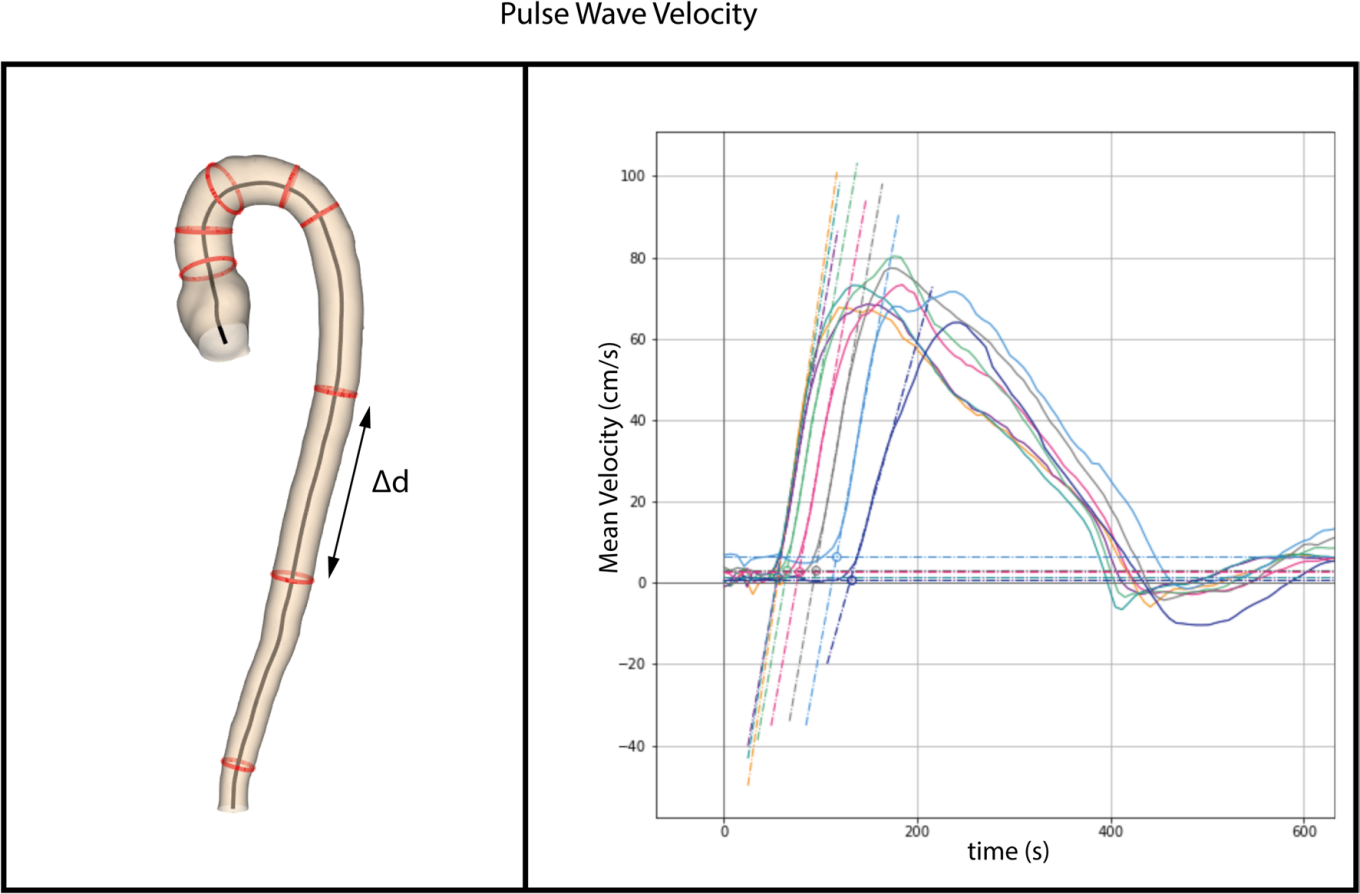
Measuring pulse wave velocity. The red circles on the aortic model (on the left) represent the planes at which flow is calculated using 4D flow data of the entire aorta. The distance between two consecutive planes (Δ*d*) is subsequently divided by the time difference (Δ*t*) between the start of respective pulse waves to yield mean pulse wave velocity (on the right)

Pulse wave velocity (PWV) is defined as the propagation speed of a pulse wave created by the systolic contraction of the left ventricle. It is a measure of arterial stiffness, which is associated with the occurrence of adverse cardiovascular events [71]. A widely accepted and often used technique for the assessment of carotid-femoral PWV is echo-Doppler. This technique is limited, however, as it cannot represent regional PWV in the aorta, and the length of the aorta is usually estimated from body length, which does not represent the true path length of the systolic wave. Another technique is conventional 2D phase contrast MRI, which has been validated against invasive pressure wave measurements (Fig. 6) [72, 73]. PWV can also be obtained from 4D flow data, and studies comparing 2D with 4D flow PWV measurements show good reliability and moderate test–retest reproducibility despite a lower temporal resolution of 4D flow MRI [74]. An advantage of 4D flow is the retrospective placement of planes, 2D flow planes having to be defined prior to image acquisition [75]. Another advantage is that 4D flow assessed PWV requires only one acquisition, where 2D phase contrast MRI assessed PWV requires repeat assessments, introducing the possibility of heart rate discrepancies.

A positive relationship has been shown to exist between increased 4D-flow PWV and presence of aortic plaques [76]. To date, no correlation has been found between changes in PWV and aortic aneurysms [77]. In patients with Marfan’s syndrome, normal PWV may be associated with absence of aortic luminal growth [78].

## Pressure fields

Transmural blood pressure, especially hypertension, is an important risk factor in cardiovascular and aortic disease. Transmural blood pressure depends on cardiac output and vascular resistance. Besides transmural pressure, pressure within a vessel may be distributed unevenly, creating local intra-vascular pressure fields. Gradients between local pressure fields (high to low) are used to assess hemodynamic significance of a vascular obstruction, such as aortic valve stenosis or aortic coarctation. Guidelines specify peak and mean gradients as important and robust parameters to assess aortic valve stenosis severity [79].

Local pressure fields can be calculated using 4D flow MRI, and requires a complex process of solving Navier–Stokes and Pressure Poisson equations [80]. Such a 4D flow MRI assessment of local pressure fields has been validated in patients with aortic coarctation using fluid structure interaction modeling [81]. Using 4D flow acquired pressure fields in the assessment of valve-related obstruction has been proven feasible in BAV patients [82, 83]. 4D flow MRI can also assess pressure gradients in an entire 3D volume, providing information on downstream pressure recovery. However, compared to other flow-related variables, 4D flow MRI acquired pressure field accuracy, reproducibility, and, therefore, clinical applicability are still relatively unknown.

## Future prospects

In current clinical practice, 4D flow imaging is mainly used in the care of congenital cardiovascular disease patients. However, there is a growing body of imaging research that suggests an influence of aberrant aortic flow on aortic dilatation. An understanding of both normal and abnormal aortic flow could therefore help identify patients at high risk of rapid aortic growth. 4D flow MRI is an appropriate tool for aortic flow evaluation, and technological advances such as decreased scanning time, automated processing and deep-learning are expected to further increase its usefulness in clinical practice.

## Data Availability

Not applicable.

## Abbreviations

2D: Two-dimensional
3D: Three-dimensional
4D: Four-dimensional
AP: Anterior–posterior
BAV: Bicuspid aortic valve
CFD: Computational fluid dynamics
CT: Computed tomography
EL: Energy loss
FD: Flow displacement
FH: Feet-head
KE: Kinetic energy
LR: Left–right
MRI: Magnetic resonance imaging
PC-MRI: Phase contrast magnetic resonance imaging
PWV: Pulse wave velocity
T: Tesla
TAA: Thoracic aortic aneurysm
TKE: Turbulent kinetic energy
US: Ultrasonography
VENC: Velocity encoding
VNR: Velocity-to-noise ratio
WSS: Wall shear stress
